# *Solanum elaeagnifolium* Var. Obtusifolium (Dunal) Dunal: Antioxidant, Antibacterial, and Antifungal Activities of Polyphenol-Rich Extracts Chemically Characterized by Use of In Vitro and In Silico Approaches

**DOI:** 10.3390/molecules27248688

**Published:** 2022-12-08

**Authors:** Mohammed Bouslamti, Amira Metouekel, Tarik Chelouati, Abdelfattah El Moussaoui, Azeddin El Barnossi, Mohamed Chebaibi, Hiba-Allah Nafidi, Ahmad Mohammad Salamatullah, Abdulhakeem Alzahrani, Mourad A. M. Aboul-Soud, Mohammed Bourhia, Badiaa Lyoussi, Ahmed Samir Benjelloun

**Affiliations:** 1 Laboratories of Natural Substances, Pharmacology, Environment, Modeling, Health and Quality of Life (SNAMOPEQ), Faculty of Sciences, Sidi Mohamed Ben Abdellah University, Fez 30000, Morocco; 2 Euromed Research Center, Euromed Faculty of Pharmacy, Euromed University of Fes (UEMF) Route de Meknes, Fez 30000, Morocco; 3 Laboratory of Biotechnology, Environment, Agri-Food and Health, Faculty of Sciences Dhar El Mahraz, Sidi Mohammed Ben Abdellah University, Fez 30050, Morocco; 4 Biomedical and Translational Research Laboratory, Faculty of Medicine and Pharmacy of the Fez, University of Sidi Mohamed Ben Abdellah, Fez 30070, Morocco; 5 Department of Food Science, Faculty of Agricultural and Food Sciences, Laval University, 2325, Quebec City, QC G1V 0A6, Canada; 6 Department of Food Science & Nutrition, College of Food and Agricultural Sciences, King Saud University, 11, P.O. Box 2460, Riyadh 11451, Saudi Arabia; 7 Department of Clinical Laboratory Sciences, College of Applied Medical Sciences, King Saud University, P.O. Box 10219, Riyadh 11433, Saudi Arabia; 8 Higher Institute of Nursing Professions and Technical Health, Laayoune 70000, Morocco

**Keywords:** *Solanum*, antimicrobial, fruits, antioxidant, leaves, HPLC-DAD, molecular docking

## Abstract

The present work was designed to study the chemical composition and the antioxidant and antimicrobial properties of fruits (SFr) and leaf (SF) extracts from *Solanum elaeagnifolium* var. obtusifolium (Dunal) Dunal (*S. elaeagnifolium).* The chemical composition was determined using HPLC-DAD analysis. Colorimetric methods were used to determine polyphenols and flavonoids. Antioxidant capacity was assessed with DPPH, TAC, and FRAP assays. Antimicrobial activity was assessed using disk diffusion and microdilution assays against two Gram (+) bacteria (*Staphylococcus aureus* ATCC-6633 and *Bacillus subtilis* DSM-6333) and two Gram (-) bacteria (*Escherichia coli* K-12 and *Proteus mirabilis* ATCC-29906), while the antifungal effect was tested vs. *Candida albicans* ATCC-1023. By use of in silico studies, the antioxidant and antimicrobial properties of the studied extracts were also investigated. HPLC analysis showed that both fruits and leaf extracts from *S. elaeagnifolium* were rich in luteolin, quercetin, gallic acid, and naringenin. Both SFr and SF generated good antioxidant activity, with IC_50_ values of 35.15 ± 6.09 μg/mL and 132.46 ± 11.73 μg/mL, respectively. The EC_50_ of SFr and SF was 35.15 ± 6.09 μg/mL and 132.46 ± 11.73 μg/mL, respectively. SFr and SF also showed a good total antioxidant capacity of 939.66 ± 5.01 μg AAE/and 890.1 ± 7.76 μg AAE/g, respectively. SFr had important antibacterial activity vs. all tested strains—most notably *B. subtilis* DSM-6333 and *E. coli*, with MICs values of 2.5 ± 0.00 mg/mL and 2.50 ± 0.00 mg/mL, respectively. SFr demonstrated potent antifungal activity against *C. albicans*, with an inhibition diameter of 9.00 ± 0.50 mm and an MIC of 0.31 ± 0.00 mg/mL. The in silico approach showed that all compounds detected in SFr and SF had high activity (between −5.368 and 8.416 kcal/mol) against the receptors studied, including NADPH oxidase, human acetylcholinesterase, and beta-ketoacyl-[acyl carrier protein] synthase.

## 1. Introduction

Medicinal plants serve as natural reservoirs of biologically active chemicals. Throughout history, people have discovered and used them for therapeutic or medicinal purposes [1]. According to the literature, almost 30% of plants are used for therapeutic purposes [2]. Medicinal plants contain various bioactive molecules, such as flavonoids, phenolic acids, tannins, carotenoids, sterols, and terpenoids [3]. These phytochemicals have shown promising microbiostatic and antimicrobial activities against a variety of pathogenic microorganisms [4,5,6]. Plants produce these compounds in response to pathogenic threats or stress conditions. Importantly, terpenoids, alkaloids, and polyphenols are involved in biological effects, including antioxidant, antibacterial, anticancer, and antidiabetic properties [7]. Due to their bioactive compounds—which are advantageous to human health against a variety of pathologies—as well as their industrial and agro-food applications, the use of medicinal plants has increased in recent years [8,9,10].

The family Solanaceae is a large angiosperm family with 96 genera and approximately 2300 species [11]. The genus *Solanum* is largely used as a drug source in medicines [12]. This genus is reported to contain steroids, saponins, alkaloids, terpenes, flavonoids, lignans, sterols, phenolic compounds, and coumarins [13]. *Solanum* spp. exhibit a wide range of pharmacological activities, including cytotoxicity against various tumors, such as colorectal, prostate, and breast cancers [14]. The species *S. elaeagnifolium* possesses anti-inflammatory, analgesic, antioxidant, and insecticidal activity, as well as hepatoprotective and anticancer activity [14,15]. Some of these biological activities may be due to its richness in quercetin, astragalin, and kaempferol 8-C-d-galactoside [4,13,14,16].

In the present work, the chemical constitution of hydroethanolic extracts from *S. elaeagnifolium* leaves and fruits was analyzed by use of HPLC-DAD as well as evaluating their antioxidant and antibacterial properties using in vitro and in silico studies.

## 2. Material and Methods

### 2.1. Plant Collection

**Figure 1:**
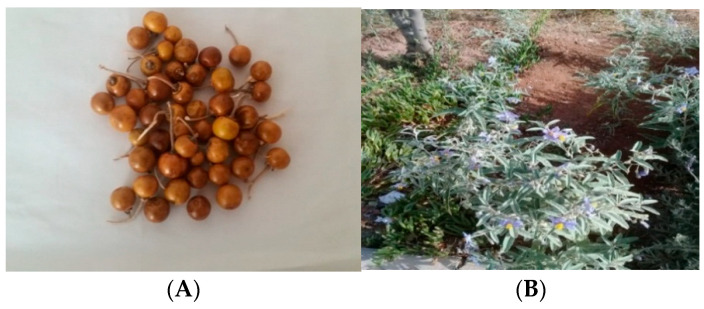
Photographs of *S. elaeagnifolium; S. elaeagnifolium* fruits (**A**) and *S. elaeagnifolium* aerial parts (**B**).

*S. elaeagnifolium* (Figure 1) was gathered during the ripening stage (end of March 2022) in the city of Fez, Morocco (34°04′04.2 N, 5°01′26.4 W). Professor Amina Bari identified the plant under E17/1405.

### 2.2. Preparation of Extract

Hydroethanolic extracts of the leaves and fruits of *S. elaeagnifolium* were prepared by maceration. In brief, 25 g of plant powder was mixed with 250 mL of ethanol (70%). The mixture was macerated for 24 h at room temperature. Finally, this mixture was filtered by use of Whatman paper. The obtained extracts were kept at 4 °C [17]. The extraction yield was calculated by the following formula:

$$Y\;\left(\%\right)=\frac{\mathrm{QE}}{\mathrm{QP}}\times 100$$

where Y (%) is the yield of the extract, QE is the quantity of the extract, and QP is the quantity of the powder.

### 2.3. Total Polyphenols

The Folin–Ciocâlteu reagent (FCR) assay was used to determine the total phenolic content in the extract. In a test tube, 0.50 mL of the extract was combined with 2.5 mL of FCR (10-fold dilution). After shaking, 4 mL of Na_2_CO_3_ (7.5%) was poured into the mixture before being incubated for 30 min at 45 °C. A UV–visible spectrophotometer was used to read the absorbance at 765 nm against a blank. A calibration curve was plotted using gallic acid as a reference standard. The results are given in milligrams of gallic acid equivalents per gram of extract (mg AGE/g Ex) [18]. 

### 2.4. Flavonoids Content

The aluminum trichloride colorimetric assay was used to determine the flavonoid content in the extract. Briefly, 1 mL of each extract was added to 5 mL of distilled water before being mixed with 0.3 mL of 5% NaNO_2_. Next, after being mixed with 0.3 mL of 10% AlCl_3_, the mixture was left to stand for 5 min at room temperature. Subsequently, 2 mL of 1 M NaOH was added to the reaction medium before the total volume was made up to 10 mL with distilled water. The whole mixture was then incubated in the shade at ambient temperature for 30 min. Afterward, the absorbance was measured at a wavelength set to 510 nm against the control by use of a UV–visible spectrophotometer. The content of flavonoids was expressed as milligrams of quercetin equivalents per 1 g of extract (mg QE/g extract) [19]. 

### 2.5. HPLC-DAD Analysis

Hydroethanolic extracts from *S. elaeagnifolium* fruit and leaves were analyzed using reverse-phase high-performance liquid chromatography (HPLC). A Thermo Scientific HPLC system with an MOS-1 HYPERSIL 250 × 4.6 mm SS Exsil ODS 5 μm analytical column was used for analysis. The separation was carried out in gradient mode with two solvents—A (water) and C (acetonitrile)—according to the following elution gradient: 80% A, 20% C for 1 min; 60% A, 40% C for 2.5 min; and 80% A, 20% C for 4 min. The flow rate was 1 mL/min, and the injection volume was 5 μL. Notably, separation was performed at a constant temperature of 40 °C, and spectrophotometric detection was performed at 254, 280, 320, 350, and 540 nm. Compounds were identified by comparing their retention times and UV spectra with those of authentic standards [17].

### 2.6. Antioxidant Capacity

#### 2.6.1. DPPH Test

Briefly, 50 μL test samples of the plant extract at various concentrations were mixed with 50 μL of a DPPH solution (0.2 mM in methanol). Next, by use of a UV–visible spectrophotometer, the absorbance was read at 517 nm after 30 min of incubation in the dark. The results are reported as a percentage of inhibition by use of the following equation [20]:

$$\mathrm{PI}\;\left(\%\right)=\frac{\mathrm{AC}-\mathrm{AE}\;}{\mathrm{AC}}\times 100$$

where AC is the uptake of the negative control, and AE is the uptake of the extract.

#### 2.6.2. FRAP Test

In summary, 0.2 mL of the extract was combined with 0.50 mL of phosphate buffer and 0.5 mL of 1% K_3_[Fe(CN)_6_]. After 20 min of incubation at 50 °C, the mixture was acidified with 0.5 mL of 10% trichloroacetic acid (TCA). Subsequently, 0.5 mL of distilled water and 0.1 mL of FeCl_3_ (0.1%) were combined with 0.5 mL of supernatant. The absorbance was determined at 700 nm [13,21].

#### 2.6.3. TAC Test

Antioxidant activity was measured by adding 1 mL of a reagent containing 0.6 M H_2_SO_4_, 28 mM Na_2_PO_4_, and 4 mM (NH_4_)_2_MoS_4_ to 100 μL of extract at various concentrations. The tubes were then tightly closed before being incubated for 90 min at 95 °C. At 695 nm, absorbance was measured after cooling. The negative control was 100 μL of methanol after 1 mL of the above reagent had been added. Under identical conditions, the samples and controls were incubated. The findings were reported in milligrams of ascorbic acid equivalents per gram (mg EAA/g) [22].

### 2.7. Antimicrobial Properties of Extracts of S. elaeagnifolium Leaves and Fruits

#### 2.7.1. Antimicrobial Activity Examination

Both the leaf (SF) and fruit (SFr) extracts of *S. elaeagnifolium* were tested for antibacterial activity using the disk diffusion assay. In brief, bacteria and *C. albicans* were inoculated into Petri dishes with Mueller–Hinton (MH) and malt extract (ME) media, respectively. Next, decimal dilutions in sterile saline were made by use of fresh cultures previously grown in MH and ME media. The final inoculum was adjusted to be 10^6^–10^8^ CFU/m. Next, the Petri dishes were inoculated with bacteria, and fungi were treated with 15 µL of 100 mg/mL SFr and SF using six-millimeter-diameter disks. To compare the effectiveness of SFr and SF, conventional antibiotics such as ampicillin for bacterial strains and the antifungal fluconazole for *C. albicans* were utilized as positive and negative controls, respectively. Thereafter, *Bacteria* and *C. albicans* were cultured in Petri dishes and incubated at 37 °C. After 1 day post-inoculation for bacteria and 2 days post-inoculation for *C. albicans*, the inhibition diameters were determined [23].

#### 2.7.2. Minimum Inhibitory Concentration (MIC) Determination

The MICs of SFr and SF against four *bacteria* and *C.albicans* were determined as previously described [23]. In summary, the microplaque was prepared in aseptic conditions; a volume of 100 μL of SFr and SF with a dilution of 1:5 (*v*/*v*) was pipetted into the first column of the plaque, before 50 μL of MH or ME was added. Next, serial dilutions were performed by use of two-fold dilution. After 1 day post-inoculation for bacteria and 2 days post-inoculation for *C. albicans*, MICs were determined by using the colorimetric method (TTC 0.2% (p/v)) [24,25].

### 2.8. In Silico Studies

Briefly, phytochemicals contained in the hydroethanolic extract of *S. elaeagnifolium*’s aerial parts were taken from the PubChem database in SDF format. Next, compounds were prepared for the docking process by use of the OPLS3 force field and the LigPrep tool of Schrödinger Software. After taking different ionization states into consideration, 32 stereoisomers were generated for each ligand (pH 7.0 2.0). 

Notably, three-dimensional crystal configurations of NAD(P)H oxidase, human acetylcholinesterase, *Escherichia coli* receptor (eta-ketoacyl-acyl carrier protein) synthase, and *Staphylococcus aureus* nucleoside diphosphate kinase were taken from the Protein Data Bank in PDB format using the PDB IDs 2CDU, 4EY7, 1FJ4, and 3Q8U, respectively. Schrödinger-Maestro v11.5’s protein preparation wizard was used to generate and construct the structures. Selenomethionines were converted into methionines, all water was removed, and heavy atoms were given hydrogens. Charges and bond ordering were also assigned, while heavy atoms’ RMSD was adjusted to 0.30 using the OPLS3 force field. By clicking on any ligand atom, the receptor grid was immediately produced. The volumetric space used was 20 × 20 × 20. In the Glide package of Schrödinger-Maestro v. 11.5, the non-cis/trans amide bond was penalized during SP flexible ligand docking. The charge cutoff was fixed at 0.150, while the van der Waals scaling factor was set to 0.80. Energy-efficient postures served as the basis for the final scoring, which was represented by the Glide score. Notably, the best-docked location with the lowest Glide score value was noted [26].

### 2.9. Statistical Analysis

All values were presented as means ± SD of triplicate experimental studies. Analysis of variance was used to determine the mean significance of the independent variables. Tukey’s multiple comparison test at *p* < 0.05 was conducted by use of GraphPad Prism 8.0.1. 

## 3. Results and Discussion

### 3.1. Analysis of the Chemical Composition of SF and SFr

The extraction of the two parts of the *S. elaeagnifolium* plant showed a difference in yields. Notably, the yield of fruits was 17.5%, while the yield of leaf extract was 11.6%. Importantly, multiple factors can influence the extraction yield, including the maceration time, harvest time, drying time, extraction solvent, and plant part used [13,27,28,29]. The extraction yield of the leaf was comparable to the value discovered by Bouslamti et al., who reported a yield of 10.2% for the hydroethanolic extract of the leaves. Feki et al. reported that the yield of the methanolic extract of *S. elaeagnifolium* seeds was 7.7% [30].

Table 1 shows the findings of the polyphenol and flavonoid evaluations. The phenolic content of the fruit extract was 0.1530 ± 0.001 mg EAG/g, while that of the leaf extract was 0.11 ± 0.01 mg EAG/g. The flavonoid content of the fruit extract was 0.015 ± 0.001 mg EQ/g, while that of the leaf extract was 0.0040 ± 0.00020 mg EQ/g. These findings are comparable to those of Bouslamti et al. [13], who discovered that the polyphenol content of the genus *Solanum* was 1.580 ± 0.030 mg EAG/g for hydroacetonic extract and 2.540 ± 0400 mg EAG/g for hydroethanolic extract. Researchers in Tunisia discovered various concentrations of polyphenols and flavonoids depending on the extraction solvent and the stages of fruit ripening [31].

**Figure 2:**
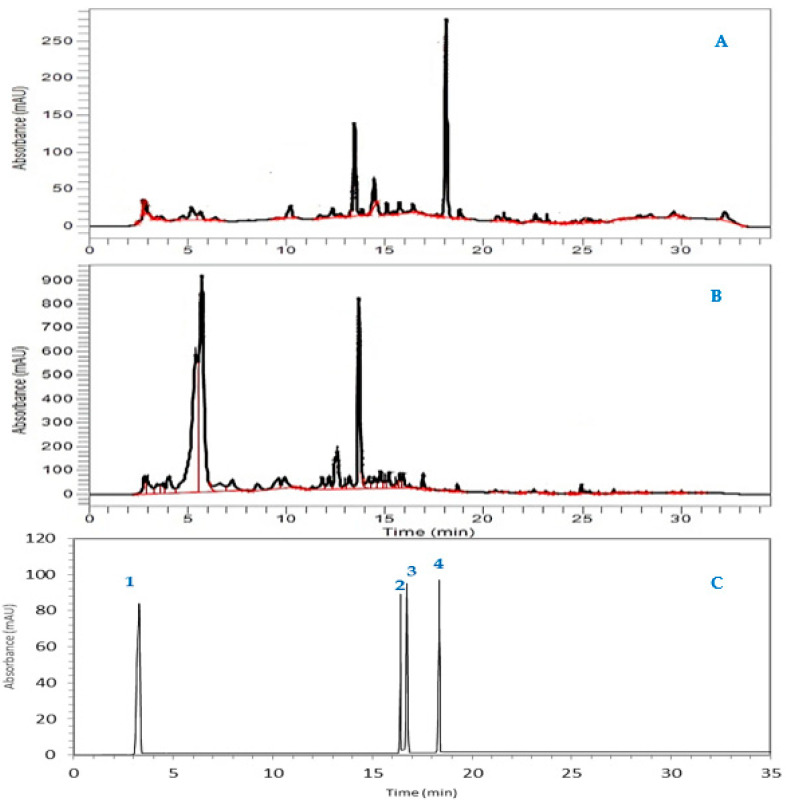
HPLC-DAD chromatogram of *Solanum elaeagnifolium* hydroethanolic extracts at 280 nm (leaves (**A**) and fruit (**B**)) and standards (**C**) (luteolin (1), quercetin (2), gallic acid (3), and naringenin (4)).

Polyphenols and flavonoids are the most bioactive secondary metabolites in plants. This chemical class may vary in terms of concentrations depending on the plant, the plant part used, the extraction method, the life cycle, and the extraction solvent used [32,33]. HPLC/DAD profile analysis of two extracts of *S. elaeagnifolium* identified four polyphenolic compounds, namely, luteolin, quercetin, gallic acid, and naringenin (Table 2; Figure 2). These findings are consistent with those reported by Al-Hamaideh and co-authors [14], who discovered that the genus *Solanum* contains phenols such as luteolin and apigenin. Notably, soil, climatic conditions, relative humidity, and postharvest treatments (e.g., drying, harvesting, and extraction) can influence phenol content variation in plants [34].

### 3.2. Antioxidant Activity

**Figure 3:**
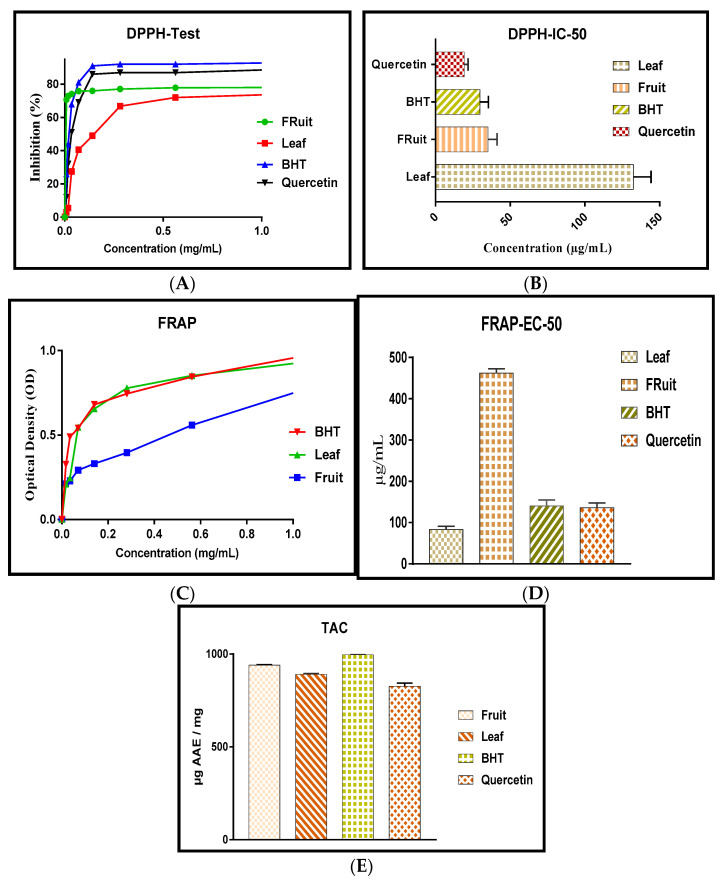
Antioxidant capacity assessed by the DPPH method: dose–effect curves (**A**) and IC_50_ (**B**). Antioxidant power assessed by the FRAP method (**C**) and EC_50_ (**D**). Total antioxidant capacity assessed by the phosphomolybdate method (**E**).

The DPPH method was used to determine the radical scavenging activity of *S. elaeagnifolium* extracts, based on the measurement of the extracts’ DPPH free radical scavenging capacity. Figure 3A shows the absorbance as a function of various concentrations of SF and SFr, while Figure 3B depicts the median inhibitory concentration (IC_50_). The IC_50_ of the fruit and leaf extracts was 35.15 ± 6.09 μg/mL and 132.46 ± 11.73 μg/mL, respectively. This activity may have been due to the presence of polyphenols and flavonoids [35,36]. 

The FRAP test results showed that both *S. elaeagnifolium* extracts had considerable reducing activity depending on the plant part utilized; notably, SF and SFr had EC_50_ values of 83.46 ± 7.69 μg/mL and 462.36 ± 10.43 μg/mL, respectively (Figure 3 C,D). The HPLC results showed the existence of phenolic compounds with a hydroxyl group with the ability to reduce iron, such as luteolin, kaempferol, apigenin, and quercetin [37,38].

The results showed that SFr had the highest total antioxidant activity of 939.66 ± 5.01 μgAAE/mg, followed by SF, which scored 890.10 ± 7.76 μg AAE/mg (3E). The antioxidant potential of the fruit and leaf extracts was found to be greater than that of quercetin. Many studies have confirmed that the genus *Solanum* has a high total antioxidant activity; notably, Bouslamti et al., who worked on the leaves of this invasive plant, discovered an antioxidant capacity of 2.54 ± 0.40 mg EAG/g for the hydroethanolic extract [13]. Rajalakshmi and Pugalenthi found that the ethanolic extract of *S. elaeagnifolium* possessed a total antioxidant activity of 19.40 mg/mL [39]. 

### 3.3. Antimicrobial Activity

**Figure 4:**
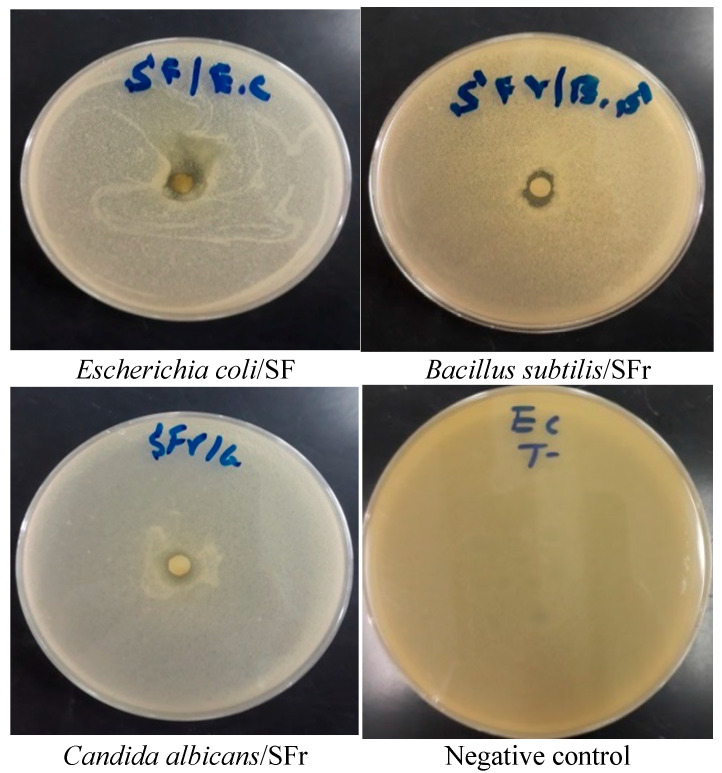
Photographs of the antimicrobial activity of *S. elaeagnifolium* leaf and fruit extracts.

The inhibitory zone diameters and MICs of SFr and SF against the tested bacterial strains and *C. albicans* are shown in Figure 4 and Table 3 and Table 4. In comparison to the antibiotic ampicillin, SFr and SF showed intriguing antibacterial activity; notably, SFr showed the highest inhibitory effect vs. *B. subtilis*, with an inhibition diameter of 10. 670 ± 0.570 mm and an MIC of 2.50 ± 0.0 mg/mL, while SF showed strong activity versus *E. coli* K12, with an inhibition diameter of 12.33 ± 0.57 mm and an MIC of 2.50 ± 0 mg/mL. Importantly, SFr showed a potent antibacterial effect versus Gram (+) bacteria, while SF showed good antibacterial activity against Gram (−) bacteria. The results showed that the SFr extract of *S. elaeagnifolium* possessed antifungal activity versus *C. albicans*, with an inhibition diameter of 9.00 ± 0.500 mm and an MIC of 0.31 ± 0 mg/mL.

Our results are consistent with those of Bouslamti and co-authors, who discovered the antibacterial activity of hydroethanolic and hydroacetonic extracts against bacteria including *E. coli* K12, *S. aureus* ATCC 6633, *B. subtilis* DSM 6333, and *Proteus mirabilis* ATCC 29,906 [13]. Extracts of the genus *Solanum* demonstrated good antibacterial effects against some clinically important pathogenic bacteria, including *Staphylococcus lentus* and *Staphylococcus haemolyticus* [40]. The chemical composition of *S. elaeagnifolium* leaf and fruit extracts is rich in secondary metabolites such as quercetin, chlorogenic acid, and apigenin [14], which contribute to its antimicrobial activity. These molecules are recognized for their bioactivity, as they possess hydroxyl groups or phenolic rings. Notably, phenolic compounds have the ability to bind to proteins and the bacterial membrane to form complexes [41].

Species of the *Solanum* genus possess antimicrobial activity, e.g., *Solanum surattense* has shown antibacterial activity against pathogenic strains—particularly *salmonella typhi* and *Shigella dysenteriae* [42,43]. The antibacterial activity observed in Solanaceae is usually related to chemicals such as terpenoids, alkaloids, and tannins that can break through the bacterial wall or the plasma membrane and exert bactericidal or bacteriostatic effects [43,44].

### 3.4. In Silico Studies

The phytocompounds identified in the hydroethanolic extracts of *S. elaeagnifolium* showed important activity. Regarding antioxidant activity, gallic acid, luteolin, quercetin, and naringenin presented Glide scores of −6.561, −6.328, −6.23, and −5.712 Kcal/mol, respectively, in the active site of NADPH oxidase. These compounds also presented Glide scores of −6.271, −8.416, −8.173, and −8.244 Kcal/mol, respectively, in the active site of human acetylcholinesterase. Regarding antimicrobial activity, gallic acid, luteolin, quercetin, and naringenin showed Glide scores of −6.756, −6.503, −5.912, and −6.703 kcal/mol, respectively, in the active site of *E. coli* beta-ketoacyl-[acyl carrier protein] synthase. Furthermore, they presented Glide scores of −5.368, −6.145, −5.95, and −6.477 Kcal/mol, respectively, in the active site of *Staphylococcus aureus* nucleoside diphosphate kinase (Table 5).

**Figure 5:**
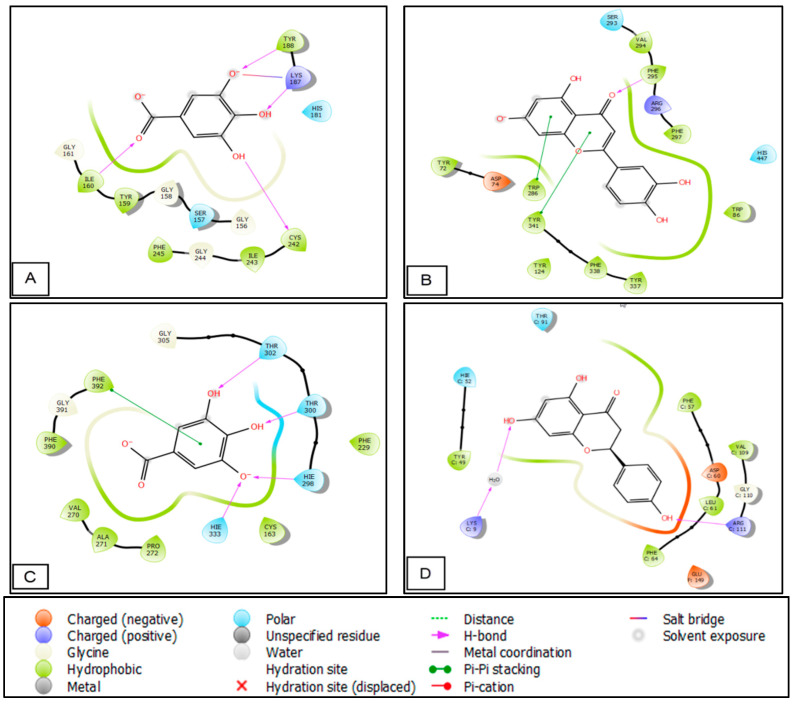
Two-dimensional (2D) view of ligands’ interaction with active sites: (**A**) interactions between gallic acid and the 2CDU active site; (**B**) interactions between luteolin and the 4EY7 active site; (**C**) interactions between the active site of 1FJ4 and gallic acid; (**D**) interactions between the active site of 3Q8U and naringenin.

**Figure 6:**
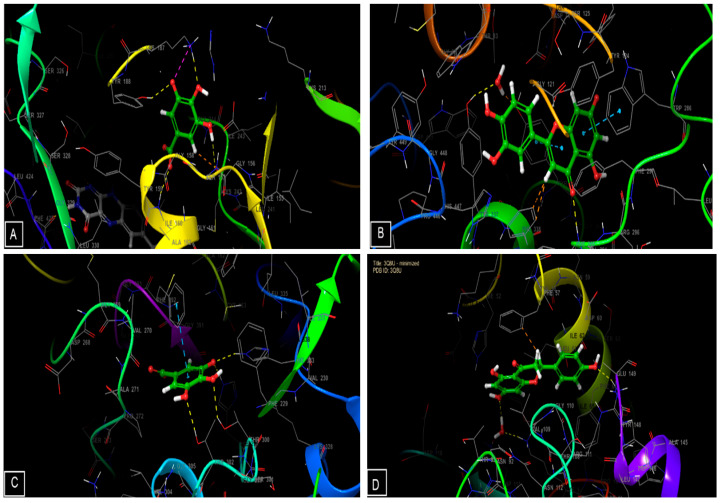
Three-dimensional (3D) view of ligands’ interaction with active sites: (**A**) interactions between gallic acid and the 2CDU active site; (**B**) interactions between luteolin and the 4EY7 active site; (**C**) interactions between the active site of 1FJ4 and gallic acid; (**D**) interactions between the active site of 3Q8U and naringenin.

Figure 5 and Figure 6 show the number and nature of possible bonds between the ligands in the active sites. In the NADPH oxidase receptor, gallic acid established four hydrogen bonds with residues TYR 188, LYS 187, CYS 242, and ILE 160, and one salt bridge with LYS187. In human acetylcholinesterase, luteolin established one hydrogen bond with residue PHE 295 and two Pi–Pi stacking bonds with residues TRP 286 and TYR 341. Concerning the antimicrobial activity, gallic acid established four hydrogen bonds with residues THR 300, THR 302, HIE 298, and HIE 333, and one Pi–Pi stacking bond with residue PHE 392 in the active site of *E. coli* beta-ketoacyl-[acyl carrier protein] synthase. Moreover, naringenin established two hydrogen bonds with residues ARG 111 and LYS 9.

## 4. Conclusions

*S. elaeagnifolium* leaf and fruit extracts were found to have antioxidant and antibacterial properties against clinical-drug-resistant bacteria in this study. Chemical analysis of *S. elaeagnifolium* extracts demonstrated the existence of four compounds—namely, gallic acid, quercetin, luteolin, and naringenin—that may be responsible for the observed activities. Fruit and leaf extracts of *S. elaeagnifolium* may be useful to help control antibacterial resistance phenomena and free-radical-related issues; however, potential adverse effects on non-target organisms need to be assessed before any use. 

## Figures and Tables

**Table 1:** Contents of phytochemical compounds in *S. elaeagnifolium* extract.

	Fruit Extract	Leaf Extract
Polyphenols	0.153 ± 0.001 mg EAG/g Ext	0.11 ± 0.01 mg EAG/g Ext
Flavonoids	0.015 ± 0.001 mg QE/g Ext	0.004 ± 0.0002 mg QE/g Ext

**Table 2:** Chromatographic analysis of phenolic compounds detected in hydroethanolic extracts of the leaves and fruit of *S. elaeagnifolium* by HPLC.

Compounds	Time (min)	Area %	
		SF	SFr
Luteolin	3.440	0.51	1.12
Quercetin	16.421	1.22	1.03
Gallic acid	16.883	0.40	0.22
Naringenin	18.781	1.93	0.97

**Table 3:** Antimicrobial properties of *S. elaeagnifolium* leaf and fruit extracts.

	*S. aureus*	*E. coli*	*B. subtilis*	*P. mirabilis*	*C. albicans*
SF	0.00 ± 0.00 ^a^	12.33 ± 0.57 ^a^	0.00 ± 0.00 ^a^	11.33 ± 0.57 ^a^	0.00 ± 0.00 ^a^
SFr	8.66 ± 0.57 ^b^	0.00 ± 0.00 ^b^	10.67 ± 0.57 ^b^	9.67 ± 0.57 ^b^	9.00 ± 0.50 ^b^
Ampicillin	8.00 ± 0.00 ^b^	10 ± 0.00 ^c^	Rs	Rs	-
Fluconazole	-	-	-	-	Rs

SFr = fruit extract; SF = leaf extract. Mean values (± standard deviation; n = 3) with different letters in the same column differ significantly (two-way ANOVA; Tukey’s test, *p* < 0.05).

**Table 4:** MICs of *S. elaeagnifolium* leaf and fruit extracts’ antimicrobial activity (mg/mL).

	***S. aureus***	***E. coli***	***B. subtilis***	***P. mirabilis***	***C. albicans***
SF	-	2.5 ± 0.00 ^a^	-	1.25 ± 0.00 ^a^	-
SFr	2.5 ± 0.00 ^a^	-	2.5 ± 0.00 ^a^	1.25 ± 0.00 ^a^	0.31 ± 0.00 ^a^
Ampicillin	5.00 ± 0.00 ^b^	5.00 ± 0.00 ^b^	-	-	-
Fluconazole	-	-	-	-	-

SFr = fruit extract; SF = leaf extract. Mean values (± standard deviation; n = 3) followed by different letters in the same column differ significantly (two-way ANOVA; Tukey’s test, *p* < 0.05).

**Table 5:** Docking results with ligands in NAD(P)H Oxidase, human acetylcholinesterase, *E. coli* beta-ketoacyl-[acyl carrier protein] synthase, and *Staphylococcus aureus* nucleoside diphosphate kinase receptors.

	2CDU		4EY7		1FJ4		3Q8U	
	Glide score (kcal/mol)	Glide energy (Kcal/mol)	Glide score (kcal/mol)	Glide energy (Kcal/mol)	Glide score (kcal/mol)	Glide energy (Kcal/mol)	Glide score (kcal/mol)	Glide energy (Kcal/mol)
Gallic acid	−6.561	−30.439	−6.271	−25.092	−6.756	−28.225	−5.368	−32.578
Luteolin	−6.328	−42.269	−8.416	−40.362	−6.503	−37.449	−6.145	−31.288
Quercetin	−6.23	−46.008	−8.173	−44.48	−5.912	−36.099	−5.95	−36.585
Naringenin	−5.712	−36.157	−8.244	−39.955	−6.703	−34.484	−6.477	−19.174

## Data Availability

Not applicable.

## Sample Availability

Samples of the compounds are not available from the authors.
